# Therapy with 2′-*O*-Me Phosphorothioate Antisense Oligonucleotides Causes Reversible Proteinuria by Inhibiting Renal Protein Reabsorption

**DOI:** 10.1016/j.omtn.2019.08.025

**Published:** 2019-09-06

**Authors:** Manoe J. Janssen, Tom T.G. Nieskens, Tessa A.M. Steevels, Pedro Caetano-Pinto, Dirk den Braanker, Melissa Mulder, Yolanda Ponstein, Shaun Jones, Rosalinde Masereeuw, Cathaline den Besten, Martijn J. Wilmer

**Affiliations:** 1 Department of Pharmacology and Toxicology, Radboud University Medical Center, Radboud Institute for Molecular Life Sciences, Nijmegen, the Netherlands; 2 Division of Pharmacology, Utrecht Institute for Pharmaceutical Sciences, Utrecht, the Netherlands; 3 BioMarin Nederland B.V., Leiden, the Netherlands; 4 BioMarin UK, London, UK

**Keywords:** Antisense oligonucleotide therapy, low molecular weight proteinuria, megalin, receptor-mediated endocytosis, proximal tubule epithelial cell

## Abstract

Antisense oligonucleotide therapy has been reported to be associated with renal injury. Here, the mechanism of reversible proteinuria was investigated by combining clinical, pre-clinical, and *in vitro* data. Urine samples were obtained from Duchenne muscular dystrophy (DMD) patients treated with drisapersen, a modified 2′*O*-methyl phosphorothioate antisense oligonucleotide (6 mg/kg). Urine and kidney tissue samples were collected from cynomolgus monkeys (*Macaca fascicularis*) dosed with drisapersen (39 weeks). Cell viability and protein uptake were evaluated *in vitro* using human conditionally immortalized proximal tubule epithelial cells (ciPTECs). Oligonucleotide treatment in DMD patients was associated with an increase in urinary alpha-1-microglobulin (A1M), which returned to baseline following treatment interruptions. In monkeys, increased urinary A1M correlated with dose-dependent accumulation of oligonucleotide in kidney tissue without evidence of tubular damage. Furthermore, oligonucleotides accumulated in the lysosomes of ciPTECs and reduced the absorption of A1M, albumin, and receptor-associated protein, but did not affect cell viability when incubated for up to 7 days. In conclusion, phosphorothioate oligonucleotides appear to directly compete for receptor-mediated endocytosis in proximal tubules. We postulate that oligonucleotide-induced low molecular weight proteinuria in patients is therefore a transient functional change and not indicative of tubular damage.

## Introduction

Single-stranded antisense oligonucleotides are a promising therapeutic platform to treat a variety of genetic diseases, cancer, and viral infections. Oligonucleotides are excreted primarily, though slowly, via the urine due to high plasma protein binding. Unbound oligonucleotides are filtered by the glomerulus, followed by reabsorption and accumulation into the proximal tubule epithelium. When exceeding critical threshold levels, oligonucleotide accumulation may become associated with tubular dysfunction in animals and man.^1^, ^2^, ^3^

Accumulation of antisense oligonucleotides in the kidney was demonstrated in animals by the presence of basophilic granules in the cytoplasm of proximal tubule epithelial cells.^4^, ^5^, ^6^ These granules are considered to reflect physiological clearance of oligonucleotides and, in the absence of associated degenerative changes, are not considered of toxicological importance.^7^, ^8^ To increase resistance to endogenous nuclease breakdown and/or target engagement, the backbone structures of antisense oligonucleotides have been chemically modified using sulfur or hydroxyl groups, resulting in phosphorothioate (PS) and 2ʹ-O-methyl (2OMe) oligonucleotides, respectively.^9^, ^10^ Dose- and concentration-dependent effects on renal tubule cells were described for the class of 2′-*O*-methoxyethylribose PS oligonucleotides. These principles likely also apply to 2OMePS antisense oligonucleotides as they share physicochemical properties.^7^, ^11^

Antisense oligonucleotides are postulated to be actively reabsorbed from the ultrafiltrate by receptor-mediated endocytosis in proximal tubule cells, as uptake of oligonucleotides is saturable.^12^, ^13^

Understanding the mechanism of uptake by proximal tubule cells and the effect of antisense oligonucleotides on kidney function is important for their safety assessment. Here, we first describe the reversible nature of the increased urinary excretion of low molecular weight proteins without overt proteinuria, observed during a long-term clinical trial of 2OMePS antisense oligonucleotide treatment in Duchenne muscular dystrophy (DMD) patients.^14^, ^15^ Next we provide supporting data obtained in monkeys and, finally, we provide mechanistic data from *in vitro* studies using the human proximal tubule cell model conditionally immortalized proximal tubule epithelial cells (ciPTECs), a cell line previously characterized in terms of intact endocytic machinery^16^, ^17^ and physiological and pharmacological characteristics,^18^, ^19^, ^20^ as well as toxicological responses.^21^, ^22^, ^23^ Together, our data provide a molecular model in which 2OMePS antisense oligonucleotides competitively inhibit receptor-mediated endocytosis in proximal tubule cells without severe cytotoxic effects or irreversible tubular dysfunction.

## Results

### Reversible Low Molecular Weight Proteinuria upon Oligonucleotide Treatment

**Figure 1:**
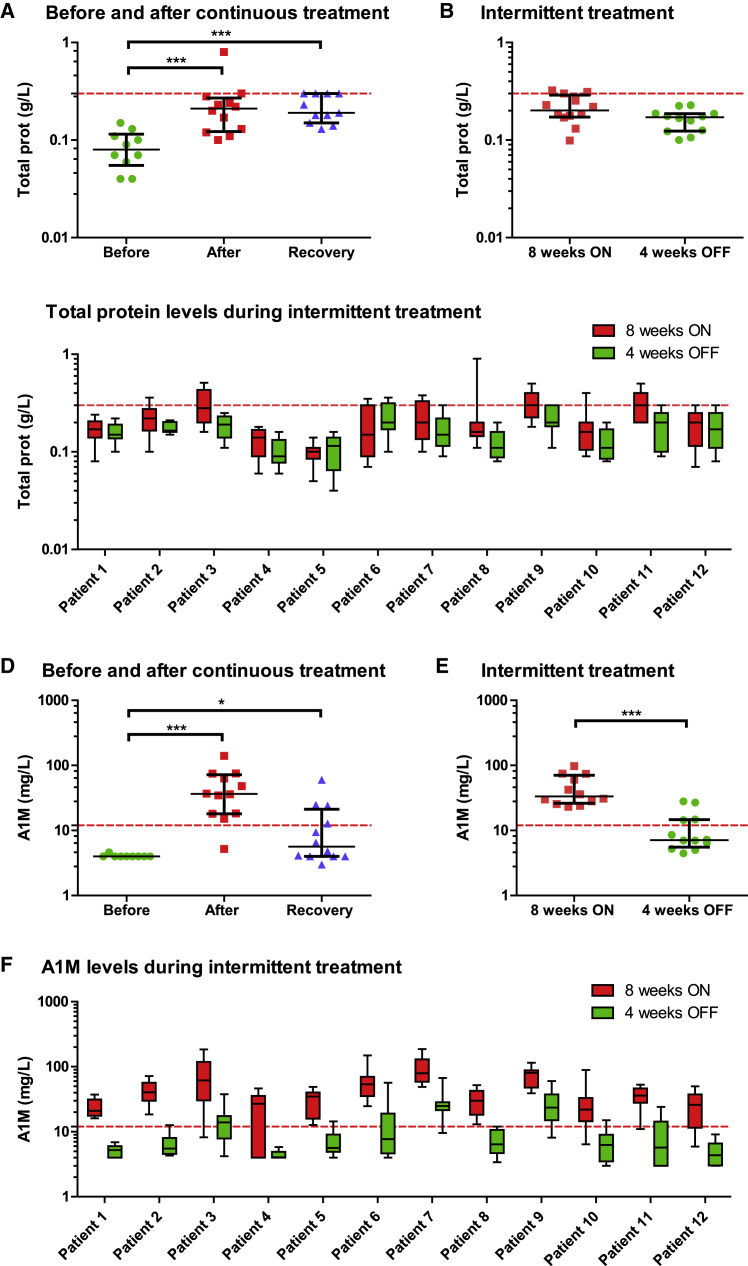
Urinary Excretion of Total Protein and A1M following Long-Term Drisapersen Treatment in DMD Patients (A–F) Total protein (A–C) and A1M (D–F) was evaluated in urine of each subject during continuous (A and D) and intermittent (B, C, E, and F) treatment with drisapersen. For the continuous treatment, drisapersen was administered weekly and urine was analyzed before the start of treatment, after 72 weeks of treatment and after an 8-week treatment interruption (recovery). During the intermittent treatment, patients underwent 9 cycles of 8 weeks on-treatment/4 weeks off-treatment. The urine values after either on- or off-treatment were pooled for each subject and grouped to show the overall effect of the intermittent treatment (B and E) as well as for each individual patient (C and F). Graphs depict the medium with the interquartile range (A, B, D, and E) or min-max values (C and F). Horizontal dashed line indicates the upper limit of normal levels in urine: 0.3 g/L for total protein and 12 mg/L for A1M. ^∗^p < 0.05, ^∗∗^p < 0.01, ^∗∗∗^p < 0.001.

Urine samples from patients were collected as part of a clinical trial in which DMD patients were treated with the antisense oligonucleotide drisapersen to change splicing of the dystrophin gene (*DMD*). Weekly subcutaneous (s.c.) administration of 6 mg/kg drisapersen for 72 weeks was associated with a slight but statistically significant increase in the concentration of both total protein and alpha-1-microglobulin (A1M) in the urine of DMD patients (Figures 1A and 1D). Median protein levels in urine increased slightly from 80 [55–115] mg/L to 210 [123–270] mg/L (p < 0.001) and remained elevated after an 8-week treatment interruption (recovery 190 [150–300] mg/L) (Figure 1A). Although the variation between and within subjects was considerable, elevations were generally mild and subclinical (i.e., urinary protein below 300 mg/L).^24^ The median levels of A1M in urine before treatment were below the detection limit of the assay (<4 mg/L) and after continuous treatment reached 36.4 [18.1–71.8] mg/L (p < 0.001). After treatment interruption, the A1M decreased toward baseline levels (5.65 [4.00–21.20] mg/L).

For the intermittent treatment, urine samples were collected for analysis before each treatment switch, and this revealed a clear pattern of reversible low molecular weight proteinuria for A1M (Figures 1D and 1E). The pooled median value after 8 weeks on-treatment (33.4 [26.2–70.8] mg/L) significantly decreased after 4 weeks off-treatment (7.11 [5.54–14.67] mg/L, p < 0.001) (Figure 1E). This pattern was also observed in individual patients (Figure 1F). Total protein did not change significantly between the on- and off-treatment cycles (Figure 1B), although a similar pattern could be seen for some individual patients (Figure 1C).

### Low Molecular Weight Proteinuria Correlates with Oligonucleotide Accumulation in Monkey Kidney

**Figure 2:**
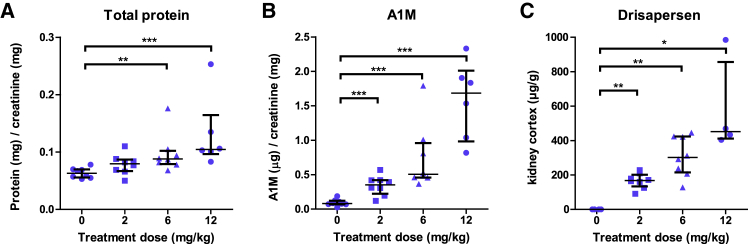
Drisapersen Treatment in Monkeys Leads to Elevated Urinary A1M and Total Protein (A–C) Urinary excretion of total protein relative to creatinine (A), urinary excretion of A1M relative to creatinine (B), and renal cortex, drug concentration of drisapersen in the renal cortex (μg/g kidney) (C) in monkeys at the end of 39 weeks dosing at 0, 2, 6, and 12 mg/kg/week by s.c. administration. Data presented as scatter dot plot with median and interquartile range corresponding to 6 to 9 (A and B) or 4 to 8 (C) male monkeys. ^∗^p < 0.05, ^∗∗^p < 0.01, ^∗∗∗^p < 0.001.

Cynomolgus monkeys treated weekly with s.c. drisapersen at 0, 2, 6, and 12 mg/kg for 39 weeks demonstrated a dose-dependent increase of A1M (i.e., median values increased 4, 6, and 21-fold over untreated controls, at doses of 2, 6, and 12 mg/kg, respectively). Urinary total protein concentrations were only slightly elevated (<2-fold). The increase in A1M correlated well (R^2^ = 0.98, p < 0.01) with the accumulation of drisapersen in the kidney cortex (Figure 2C). Histopathological evaluation of the kidney did not reveal any tubular abnormalities apart from a dose-related increase of the antisense oligonucleotide manifested as the presence of basophilic granules in proximal tubules (data not shown).

### Endocytic Uptake and Limited Degradation of Oligonucleotides in Proximal Tubular Cells *In Vitro*

**Figure 3:**
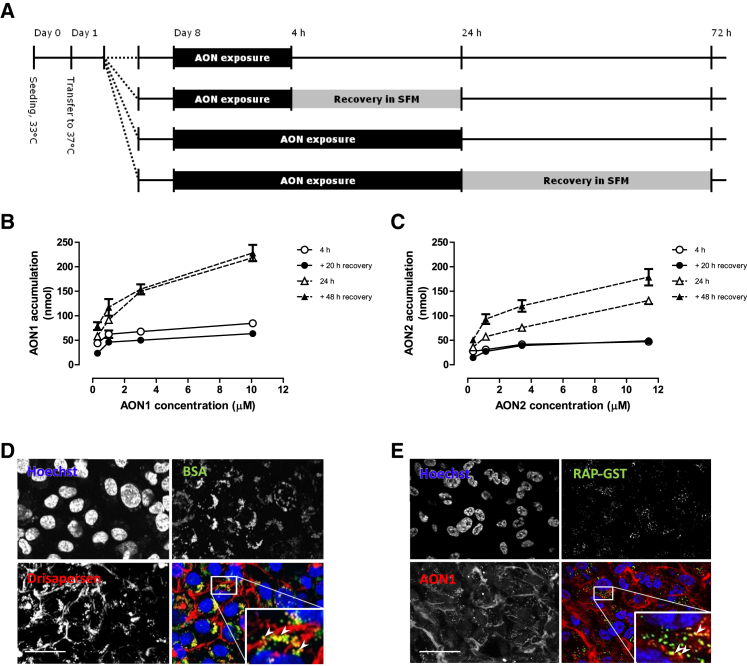
Uptake of Antisense Oligonucleotide by ciPTECs (A) Schematic overview of the different AON incubation and recovery times in ciPTECs (B and C). Accumulation of AON inside ciPTECs when exposed for 4 h or 24 h to different concentrations of AON1 (0.32–10.8 μM) (B) or AON2 (0.34–11.4 μM) (C). The ciPTECs were harvested immediately or after a recovery period of respectively 20 h or 48 h in the absence of AON (n = 1). (D and E) Uptake after 24 h of fluorescently labeled drisapersen (in red) with BSA (green) (D), or fluorescently labeled AON1 (red) with RAP-GST (green) (E), nuclei are stained blue (Hoechst). Data are presented as mean ± SD, and scale bar is 40 μm.

To evaluate uptake of antisense oligonucleotides *in vitro*, we incubated human ciPTECs with drisapersen, antisense oligonuceotide 1 (AON1), or antisense oligonuceotide 2 (AON2) (oligonucleotides with similar 2OMePS chemistry to drisapersen but with a different nucleotide sequence) (Figure 3A). Incubation at different time points (4 and 24 h) and different concentrations (ranging from 0.3 to 10× C_max_ of drisapersen in clinical trials)^25^ showed a time- and concentration-dependent uptake of AON in ciPTECs (Figures 3B and 3C). Recovery for 20 or 48 h following AON uptake did not reduce levels in the cell, indicating that the intracellular antisense oligonucleotides are not sensitive to degradation within this time period. Confocal microscopy using fluorescently labeled AONs (Figures 3D and 3E) demonstrated that the AONs accumulate in a vesicular pattern, suggesting sequestration in endosomes. Co-localization with fluorescently labeled albumin (BSA-Alexa 657) (Figure 3D) or GST-tagged receptor-associated protein (RAP-GST) (Figure 3E), two known ligands of receptor-mediated endocytosis, confirmed that the uptake of AONs in tubule cells occurs via the same endocytotic pathway.

### Long-Term Exposure to Antisense Oligonucleotides Does Not Result in Cytotoxicity

**Figure 4:**
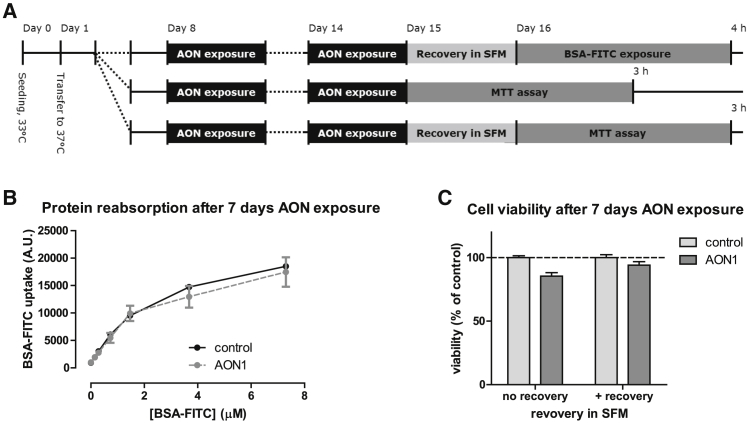
Intact ciPTEC Functionality upon Long-Term Exposure to Antisense Oligonucleotides (A) Schematic overview of ciPTEC pre-incubation with AON1, recovery and evaluation of BSA-FITC uptake capacity, and MTT viability assay. (B) BSA-FITC (0.15–7.3 μM) uptake by ciPTECs for 4 h at 37°C when pre-incubated with AON1 (1.15 μM) for 7 days and subsequently recovered for 24 h, as evaluated by multiwell platereader (n = 2). (C) Viability of ciPTECs after exposure to AON1 (1.15 μM) for 7 days, with and without recovery, relative to cell viability without exposure (n = 2).

To evaluate long-term adverse effects of renal tubule cell exposure to antisense oligonucleotides, we pre-incubated ciPTECs with AON1 at C_max_ levels for 7 days, followed by 24 h recovery in absence of AON (Figure 4). Next, the BSA uptake assay was performed in absence of AON to evaluate whether long-term AON incubation had reduced the overall protein reabsorption capacity of these cells. This showed that the 7-day pre-incubation with AON1 did not affect BSA uptake by ciPTECs (Figure 4B). Also no changes in cell viability were found, both with and without 24 h recovery (Figure 4C).

### Antisense Oligonucleotides Compete with Protein Reabsorption *In Vitro*

**Figure 5:**
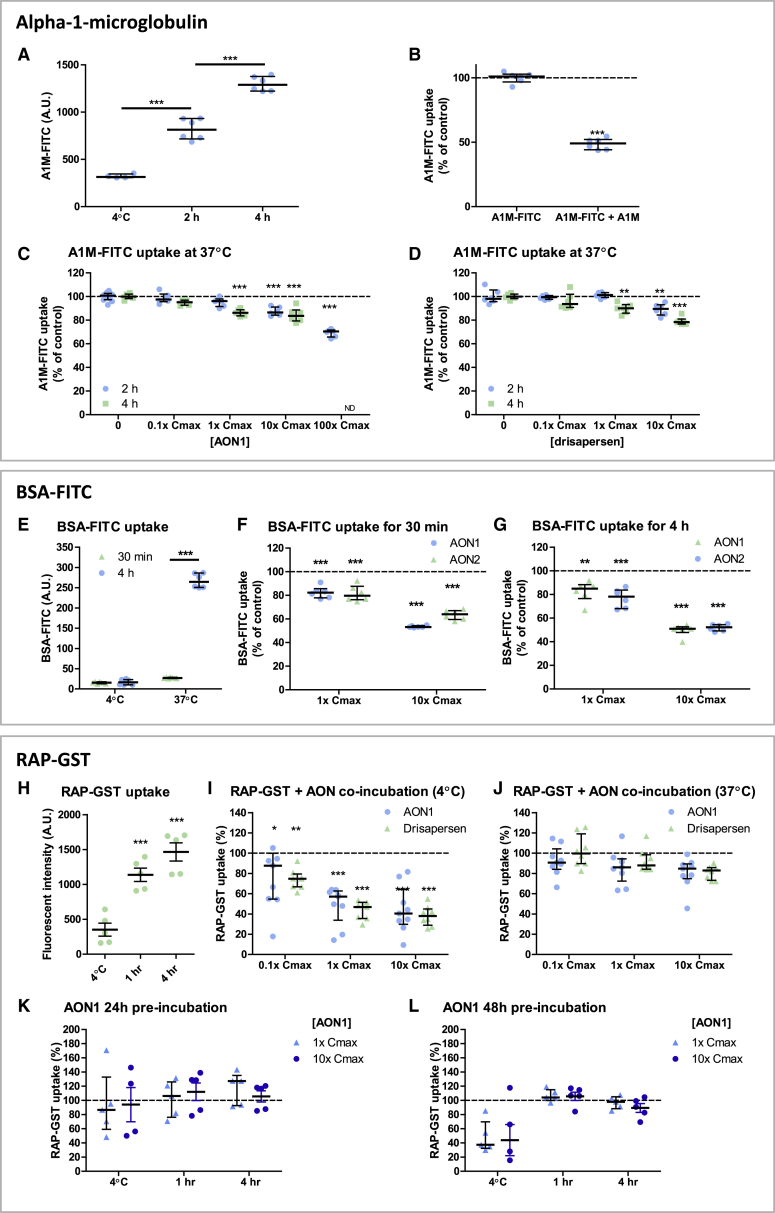
Protein Reabsorption in Proximal Tubular Cells Is Inhibited by AON (A) A1M-FITC uptake by ciPTECs, for 2 h at 4°C, and for 2 h and 4 h at 37°C (n = 2). (B) A1M-FITC uptake by ciPTECs incubated for 2 h at 37°C in the presence or absence of an equal concentration of unlabeled A1M (3.85 μM) (n = 2). (C and D) A1M -FITC (3.85 μM) uptake by ciPTECs when co-incubated with different concentrations of (C) AON1 (0.11–108 μM) or (D) drisapersen (0.14, 1.35, and 13.5 μM) for 2 h and 4 h at 37°C, relative to uptake without antisense oligonucleotide, as evaluated by flow cytometry (n = 2). (E) Fluorescent intensity of ciPTECs following incubation with BSA-FITC at 4°C or 37°C for either 30 min (F) or 4 h (G) (n = 2). BSA-FITC uptake by ciPTECs when co-incubated with AON1 or AON2 for 30 min or 4 h at 37°C, as evaluated by flow cytometry (n = 2). (H) RAP-GST cell surface binding (4°C for 30 min) and active uptake in proximal tubular cells (37°C at 1 and 4 h) in absence of antisense oligonucleotide. (I and J) Co-incubation with either AON1 or drisapersen inhibited the cell surface binding of RAP-GST (30 min at 4°C) (I) and the active uptake of RAP-GST (4 h at 37°C) (J) in a concentration-dependent manner. (K and L) Cells were pre-incubated with AON1 for 24 h (K) and 48 h (L) prior to analyzing RAP-GST endocytic capacity in absence of AON1. ND, not determined. *p < 0.05, **p < 0.01, ***p < 0.001.

The reversible low molecular weight proteinuria observed in patients may be the result of competition for receptor-mediated endocytosis rather than a consequence of direct tubular toxicity. The receptor proteins megalin and cubilin at the apical membrane of the proximal tubule mediate reabsorption by binding and uptake of ligands, including A1M,^26^, ^27^ receptor associated protein (RAP), and albumin. RAP (gene name *LRPAP1*) is a 39 kDa chaperone protein that binds to megalin with high affinity,^28^, ^29^, ^30^ whereas albumin is a large ∼68 kDa plasma protein that in small amounts can pass the glomerular filter and is efficiently reabsorbed by the proximal tubule.^31^, ^32^ We therefore examined the uptake of fluorescently labeled A1M (A1M-FITC) (Figures 5A–5D), fluorescently labeled BSA-FITC (Figures 5E–5G), and RAP-GST (Figures 4H–4L) in ciPTECs in the absence or presence of antisense oligonucleotides. At 37°C, A1M-FITC was actively taken up over time by ciPTECs (Figure 5A) and could be inhibited by excess unlabeled A1M (Figure 5B). Antisense oligonucleotides (AON1 or drisapersen) reduced A1M-FITC uptake in ciPTECs in a time- and concentration-dependent manner (Figures 5C and 5D), suggesting competitive interaction with the same receptor.

Co-incubation with AON1 or AON2 significantly inhibited BSA-FITC uptake (up to 50%) in ciPTECs at both 30 min and 4 h in a concentration-dependent manner (Figures 5E–5G). The presence of antisense oligonucleotides also interfered with RAP-GST cell surface binding at 4°C (up to 70% reduction in binding) and inhibited active uptake of RAP-GST at 37°C (up to 25% reduction) (Figures 5H–5J).

Pre-incubation with oligonucleotides for 24 or 48 h prior to incubation with the labeled substrate did not significantly inhibit the uptake of RAP-GST at 37°C (Figures 5H and 5I), indicating that antisense oligonucleotide incubation directly competes with receptor binding capacity and has no cytotoxic effect that affects the megalin- or cubilin-mediated endocytosis rate. Some residual inhibition was seen at 4°C after pre-incubation, which is likely due to occupation of ligand binding sites at the cell surface that were not washed away at 4°C.

Together these data indicate that antisense oligonucleotides directly compete with receptor-mediated protein reabsorption in ciPTECs, without adversely affecting the reabsorption capacity of the cells.

## Discussion

Antisense oligonucleotide therapeutics accumulate dose dependently in renal proximal tubular cells, and renal tubular effects are commonly associated with oligonucleotide treatment in both animal studies and in the clinic.^1^, ^3^, ^4^, ^7^ Little is known, however, about the cellular mechanism and the potential adverse effects. In this study, we investigated how 2OMePS oligonucleotides affect urinary protein excretion, epithelial reabsorption, and cytotoxicity by combining clinical, pre-clinical, and *in vitro* data. Our data support the hypothesis that 2OMePS oligonucleotides compete with receptor-mediated endocytosis in the proximal tubule epithelium and reduce the uptake of endogenous ligands such as A1M, resulting in reversible low molecular weight proteinuria in patients and animals.

Low molecular weight proteins pass the glomerular filter and are prevented from excretion through active tubular reabsorption. The presence of A1M in urine is, therefore, often considered indicative of impaired tubular function.^33^ Our clinical data show that the levels of A1M in urine correlated strongly to antisense oligonucleotide therapy in DMD patients, but normalized during planned off-treatment periods. Total protein remained well below levels associated with renal disease, suggesting reversible proximal tubule function impairment rather than cytotoxicity. Of note, urinary protein was not normalized to urinary creatinine, because this is highly dependent on skeletal muscle mass and unsuitable as a reference in DMD patients.^34^ Although the level of oligonucleotide accumulation in renal tissue from patients is not known, the tubular effects are thought to be closely linked to the degree of uptake into proximal tubular cells.^3^, ^6^ This was also seen in the monkeys where drisapersen accumulation in the kidney closely correlated with the urinary protein (without any histopathological evidence of tubular toxicity after 39 weeks of treatment). Elimination of oligonucleotides is known to follow first-order kinetics; after a steady state is reached, the oligonucleotide concentration in cells does not increase further with increasing duration of treatment.^7^ The kinetics of tissue uptake and elimination are primarily determined by the PS backbone of the oligonucleotide, but species differences challenge prediction of tubular accumulation in humans.^35^ However, when patients were treated with drisapersen for 72 weeks, levels of A1M and total protein increased in the first weeks of treatment and remained stable thereafter, reflecting steady-state kinetics. It is also important to note that the reversible effects in the tubular compartment described in this paper are distinct from glomerular effects that have occasionally been seen with PS antisense oligonucleotides.^7^, ^36^ Glomerular effects are related to the inflammatory potential of oligonucleotides, for which animals are thought to be more sensitive than humans.

In the current study, antisense oligonucleotides accumulated in human proximal tubule cells *in vitro* without cytotoxic effects after 7 days of exposure to antisense oligonucleotide at concentrations equivalent to or exceeding peak plasma levels of drisapersen in humans. Consistent with the long tissue half-life of 2OMePS oligonucleotides (estimated in humans to be over 5 weeks),^35^ no significant degradation was seen after 20 h or 48 h recovery. To investigate the link between oligonucleotide treatment and proteinuria, we evaluated receptor-mediated protein reabsorption by proximal tubule cells *in vitro*, using the well-characterized ciPTEC model. This cell line is human-derived and has demonstrated tubular epithelial features, including functional basolateral and apical drug transporters such as organic cation transporter 2, P-glycoprotein 1, and multidrug resistance proteins, as well as intact receptor-mediated endocytosis, including expression of megalin and cubilin.^16^, ^17^, ^18^, ^19^, ^20^, ^21^, ^22^, ^23^ Dysfunction of proximal tubular protein reabsorption by megalin and cubilin causes low molecular weight proteinuria in both animal models and human disease,^37^ and as we hypothesize, so does competitive inhibition via saturation by oligonucleotide treatment.

A1M and, to a lower extent, albumin are both present in the glomerular filtrate and are almost completely reabsorbed by receptor-mediated endocytosis in proximal tubule cells. Incubation of ciPTECs with antisense oligonucleotides indeed reduced the binding and uptake of A1M, albumin, and RAP, whereas pre-incubation with antisense oligonucleotide for 24 h and up to 7 days did not diminish protein reabsorption. This suggests a direct competition at the receptor level, rather than a reduction in reabsorption capacity due to intracellular oligonucleotide accumulation. The oligonucleotides may block protein receptor binding sites at the cell surface, or directly interact with A1M, albumin, and RAP in the solution that can prevent their binding and internalization. Both hypotheses would explain the reduced uptake of proteins from the filtrate and their release in the urine.

In this study we focused on the effect of oligonucleotide exposure on protein reabsorption and cell viability in proximal tubule cells. Although we find that the 2OMePS oligonucleotides interfere with protein uptake, this does not directly imply that the proteins can also block oligonucleotide uptake, or that the bulk of oligonucleotides are taken up by the same pathway as the protein ligands. So far, several different pathways have been described for the uptake of PS-modified oligonucleotides, which vary greatly among different tissues and organisms and can even depend on the oligonucleotide sequence.^5^, ^38^, ^39^, ^40^, ^41^, ^42^ To be therapeutically active following endocytosis in the tubule cells, the oligonucleotides have to reach the cytoplasm or nucleus without degradation in the endo-lysosomal compartment. Although the receptors and pathways involved have not been fully elucidated, a distinction is generally made between a productive pathway (oligonucleotides reach their target and lead to gene knockdown or alternative splicing) or a nonproductive pathway (oligonucleotides stay in the endo-lysosomal compartment and are degraded).^38^, ^40^

The majority of PS-modified oligonucleotides are protein bound in the blood (>90%), which facilitates its uptake into cells and tissues and reduces the free fraction available for glomerular filtration.^43^ This raises the question whether *in vivo* proximal tubule cells will encounter the oligonucleotides mostly in its unbound or protein-bound form, because this may change the uptake route and interaction with membrane proteins. Animal studies have also shown that PS-modified oligonucleotides can enter proximal tubule cells from both the tubular and the capillary side, and different uptake mechanisms may be involved in each case.^44^ Here the *in vitro* oligonucleotide uptake experiments (Figures 3B and 3C) have been performed in serum-free medium, so uptake has taken place in the absence of serum proteins like albumin. We also evaluated whether coincubation with BSA, A1M, or RAP-GST could block or enhance oligonucleotides uptake, but the presence of these ligands did not affect oligonucleotide uptake over 24 h (data not shown). This suggests that oligonucleotide uptake predominantly takes place in its free form and uptake is not facilitated by binding to serum proteins like albumin.

In conclusion, our data support a model of direct competition of 2OMePS antisense oligonucleotide with protein reabsorption in proximal tubule cells, consistent with the observations of reversible proteinuria in patients and the absence of tubular damage in monkeys. This supports the limited risk for kidney tubular injury at therapeutic doses of PS oligonucleotides.

## Materials and Methods

### Compounds

The oligonucleotides used in this study have a fully modified 2OMePS backbone and were synthesized at BioMarin Nederland B.V. (Leiden, the Netherlands). Sequences of drisapersen (PRO051), AON1, and AON2 are 5′-UCA AGG AAG AUG GCA UUU CU-3′ (targets dystrophin exon 51), 5′-UAU GAG UUU CUU CCA AAG CAG CCU C-3′ (5′-methylated Cs) and 5′-GAG UUU CUU CCA AAG CAG CCU CUC-3′, respectively. AON1-Texas red and drisapersen-Cy3 were synthesized by Biospring (Frankfurt, Germany) and BioMarin Nederland, respectively.

### Urinalysis in Human Subjects

Urine samples were collected during an open-label study in 12 DMD patients receiving drisapersen (DMD114673).^14^ Treatment involved weekly s.c. administration at 6 mg/kg body weight for 72 weeks, followed by an 8-week treatment interruption, and subsequently 9 intermittent treatment cycles of 8 weeks on-treatment/4 weeks off-treatment. First morning urine was used for total protein and A1M analysis, before or 1 week after treatment. Excretion was not normalized to creatinine because DMD patients have reduced muscle mass.^24^, ^45^

### Study in Monkeys

Cynomolgus monkeys (*Macaca fascicularis*) received drisapersen (in 20 mM phosphate buffer with 0.8% w/v NaCl) for 39 weeks by weekly s.c. administration at dose levels of 0, 2, 6, and 12 mg/kg body weight. Overnight urine was collected and analyzed for total protein and creatinine using ADVIA 1650 (Siemens), and A1M using Myriad RBM’s Human CustomMAP. At necropsy (39 weeks) kidney cortex was snap-frozen for drisapersen analysis by hybridization-ligation assay as described previously.^46^ The *in vivo* study was ethically reviewed and carried out in accordance with European Directive 86/609/EEC.

### Cell Culture

Development of the ciPTECs used in this studied is described in detail elsewhere.^20^ In brief, primary renal cells were isolated from the urine of a healthy volunteer and immortalized by transduction with human telomerase (hTERT) and the temperature sensitive SV40 large T antigen (SV40T) to promote respectively genome stability and cell expansion at 33°C. In this way, a stable clone exhibiting robust proximal tubule characteristics was selected and further characterized based on the presence of an intact endocytic machinery and physiological and pharmacological characteristics, as well as toxicological responses.^16^, ^17^, ^18^, ^19^, ^20^, ^21^, ^22^, ^23^ Cells were seeded 7 days before the experiment at 55,000 cells/cm^2^ and grown for 1 day at 33°C and 5% v/v CO_2_, followed by 6 days at 37°C and 5% v/v CO_2_ to confluency in culture medium as previously described^47^ to stimulate receptor-mediated endocytosis. Experiments are performed in complete medium (containing DMEM HAM’s F12, Life Technologies, Paisley, UK), 5 μg/mL insulin, 5 μg/mL transferrin, 5 μg/mL selenium, 35 ng/mL hydrocortisone, 10 ng/mL epidermal growth factor (EGF), 40 pg/mL tri-iodothyronine (Sigma, St. Louis, MO, USA), and 10% fetal calf serum (FCS, Greiner Bio One, Kremsmuenster, Austria), or in culture medium without FCS, named “serum-free medium” (SFM).

### Antisense Oligonucleotide Uptake

Cells were washed (HBSS, Invitrogen, Carlsbad, CA, USA) and exposed to 0.32–10.8 μM AON1 or 0.34–11.4 μM AON2 for 4 h or 24 h at 37°C. Samples were either harvested immediately or subsequently incubated in SFM to recover for 20 h (4 h exposure) or 48 h (24 h exposure). Cells were harvested by washing in HBSS, heparin (20 U/mL, Sigma, St. Louis, MO, USA), and HBSS, before lysation in RLT buffer (1 min, room temperature; QIAGEN, Hilden, Germany). Lysates were evaluated as described previously.^27^

### A1M-FITC and BSA-FITC Uptake Determination by Flow Cytometry

Cells were washed and pre-incubated with SFM for 24 h at 37°C 5% v/v CO_2_. On day 8 after seeding, cells were washed (SFM) and co-incubated with AON1 or AON2 (0.11, 1.08, 10.8, or 108 μM and 0.11, 1.14, or 11.4 μM respectively in SFM) or drisapersen (0.14, 1.35, or 13.5 μM), and BSA-FITC (0.73 μM) or A1M-FITC (3.85 μM; Α1Μ from https://www.antibodies-online.com/ conjugated to FITC from Life Technologies) for 30 min, 2 h, or 4 h at 37°C. A control set using BSA-FITC or A1M-FITC only was additionally incubated at 4°C. Next, cells were washed (HBSS and harvested using trypsin-EDTA, Invitrogen, Carlsbad, CA, USA). The cell suspension was washed (HBSS), fixed using 0.5% paraformaldehyde, and analyzed on a FACS Calibur (Becton Dickinson, Franklin Lakes, NJ, USA).

### RAP-GST Binding and Uptake

Cells were incubated with medium containing 0.38 μM RAP-GST at 4°C for cell surface binding or at 37°C for active protein uptake. Cells were washed (medium), fixed in 4% PFA for 10 min, washed (PBS), and permeabilized for 5 min using 0.1% Triton X-100 in PBS. Cells were washed (PBS) and incubated overnight with primary anti-GST antibody in 1% BSA/PBS (1:1,600, GE Healthcare, 27457701), followed by 2 h incubation with secondary ab (1:500, Alexa Fluor 488), washed (PBS), and quantified by plate reader (Ex 485 nm; Em 535 nm) or using fluorescence microscopy.

### Antisense Oligonucleotide Fluorescent Imaging

Cells were incubated for 4 and 24 h with AON1-Texas red or drisapersen-Cy3, in absence or presence of BSA-Alexa Fluor 647 (Thermo Fisher Scientific A34785, 5 μg/mL) or RAP-GST (recombinant protein produced in house, 2.5 μg/mL). Cells were washed and, when incubated with drisapersen-Cy3 and BSA-Alexa Fluor 647, imaged live, whereas cells incubated with AON1-Texas red and RAP-GST were first fixed and permeabilized (before incubation with anti-GST-ab, GE Healthcare 27457701, goat polyclonal, overnight 1:1,600 in 1% BSA/PBS) and stained with secondary ab (Alexa Fluor 488, donkey anti goat, 1:500 in 1% BSA/PBS). Images were taken with a Yokogawa CV7000 confocal imager and analyzed using ImageJ.

### Functional Uptake Assay upon Long-Term Pre-Incubation

Cells were exposed to 1.08 μM AON1 in complete culture medium for 7 days at 37°C 5% v/v CO_2_, refreshed daily. Cells were washed (SFM) and incubated in absence of antisense oligonucleotides for 24 h. BSA-FITC uptake capacity was evaluated by washing cells (SFM) and incubation with 0.15–7.3 μM BSA-FITC in SFM for 4 h at 37°C. After washing 3 times with ice-cold HBSS (Invitrogen, Carlsbad, CA, USA), fluorescence was quantified by plate reader (ex 485 nm; em 535 nm).

### Viability Assay upon Long-Term Pre-Incubation

Cells were exposed to 1.08 μM AON1, refreshed daily. Cells were washed (SFM) and either first incubated in SFM in absence of antisense oligonucleotides for 24 h before the tetrazolium dye MTT (3-(4,5-dimethylthiazol-2-yl)-2,5-diphenyltetrazolium bromide) (Sigma) was added (0.5 mg/mL, 3 h), or the MTT assay was performed immediately. Formazan was dissolved in DMSO (Merck, Whitehouse Station, NJ, USA) and optical density was measured (560 nm, background at 670 nm was subtracted) using Benchmark Plus (Bio-Rad, Hercules, CA, USA).

### Data Analysis and Statistics

For antisense oligonucleotide uptake and inhibition assays, data are either presented as lysate concentration or normalized to samples without inhibitor. For A1M-FITC, BSA-FITC, and RAP-GST uptake and inhibition assays, data are either presented as absolute fluorescence values or normalized to the activity of samples without antisense oligonucleotide and plotted after background subtraction. For MTT viability assay, data were normalized to the viability of control cells. Statistics was performed by one or two-way ANOVA (two-tailed, α = 0.05) using GraphPad Prism. All data are plotted with GraphPad Prism (version 6.07) and are presented as mean ± SD of at least two separate experiments (n = 2) performed in triplicate, unless stated otherwise.

## Author Contributions

Conceptualization, M.J.W., R.M., and C.d.B.; Methodology, M.J.J., T.T.G.N., M.J.W., T.A.M.S., and M.M.; Validation, M.J.J., T.T.G.N., T.A.M.S., and M.M.; Formal Analysis, M.J.J., T.T.G.N., A.M.S., and M.M.; Investigation, M.J.J., T.T.G.N., T.A.M.S., P.C.-P., and D.d.B.; Writing – Original Draft, M.J.J. and T.T.G.N.; Writing – Review and Editing, Y.P., S.J., C.d.B., T.A.M.S., M.J.W., and R.M.; Visualization M.J.J.; Supervision, C.d.B., M.J.W., and R.M.; Project Administration, M.J.W.; Funding Acquisition, M.J.W., R.M., and C.d.B.

## Conflicts of Interest

T.A.M.S., M.M., and S.J., are and Y.P. and C.d.B. were employees of BioMarin Nederland B.V. S.J. is current employee of BioMarin UK Limited. M.J.W. is co-inventor on patent EP2010/066792 “Novel conditionally immortalized human proximal tubule cell line expressing functional influx and efflux transporters” assigned to Radboudumc and as such M.J.W. has a conflict of interest through commercialization of ciPTEC models via Cell4Pharma. T.T.G.N. is currently an employee of AstraZeneca AB, Gothenburg, Sweden. All other authors declare no competing interests.
